# Genomic characterization of *Listeria monocytogenes* isolates reveals that their persistence in a pig slaughterhouse is linked to the presence of benzalkonium chloride resistance genes

**DOI:** 10.1186/s12866-018-1363-9

**Published:** 2018-12-20

**Authors:** Tamazight Cherifi, Catherine Carrillo, Dominic Lambert, Ilhem Miniaï, Sylvain Quessy, Guillaume Larivière-Gauthier, Burton Blais, Philippe Fravalo

**Affiliations:** 1 Chaire de Recherche en Salubrité des Viandes, Faculté de Médecine Vétérinaire, Université de Montréal, Saint-Hyacinthe, QC Canada; 2 Centre de Recherche sur les Maladies Infectieuses Porcine et Avicole, Faculté de Médecine Vétérinaire, Université de Montréal, Saint-Hyacinthe, QC Canada; 3 Groupe de Recherche et D’enseignement En Salubrité Des Aliments, Faculté de Médecine Vétérinaire, Université de Montréal, Saint-Hyacinthe, QC Canada; 4 Food Microbiology Research Team, Canadian Food Inspection Agency, Ottawa, ON Canada

**Keywords:** *bcrABC* cassette, Benzalkonium chloride resistance genes, *Listeria monocytogenes*, Pig slaughterhouse, Whole genome sequences

## Abstract

**Background:**

The aim of this study was to characterize the genomes of 30 *Listeria monocytogenes* isolates collected at a pig slaughterhouse to determine the molecular basis for their persistence.

**Results:**

Comparison of the 30 *L. monocytogenes* genomes showed that successive isolates (i.e., persistent types) recovered from thew sampling site could be linked on the basis of single nucleotide variants confined to prophage regions. In addition, our study revealed the presence among these strains of the *bcrABC* cassette which is known to produce efflux pump-mediated benzalkonium chloride resistance, and which may account for the persistence of these isolates in the slaughterhouse environment. The presence of the *bcrABC* cassette was confirmed by WGS and PCR and the resistance phenotype was determined by measuring minimum inhibitory concentrations. Furthermore, the BC-resistant strains were found to produce lower amounts of biofilm in the presence of sublethal concentrations of BC.

**Conclusions:**

High resolution SNP-based typing and determination of the *bcrABC* cassette may provide a means of distinguishing between resident and sporadic *L. monocytogenes* isolates, and this in turn will support better management of this pathogen in the food industry.

**Electronic supplementary material:**

The online version of this article (10.1186/s12866-018-1363-9) contains supplementary material, which is available to authorized users.

## Background

*Listeria monocytogenes* is a bacterial pathogen that causes listeriosis, the foodborne illness with the highest fatality rate [1, 2]. The ability of this organism to colonize and persist in food processing plants increases the risk of food contamination [3]. Persistent strains have been involved in some listeriosis outbreaks [4–6], making persistent contamination of *L. monocytogenes* an important consideration for public health. The importance of the persistence of *L. monocytogenes* in food processing plants has been previously discussed [7–9]. Whereas some *L. monocytogenes* strains can persist within the food processing environment for years [3], some strains are merely transitory in these environments, and the approaches for their mitigation will be very different (i.e., different sanitation regimens). Many genetically distinct *L. monocytogenes* strains can be isolated sporadically during different sampling periods while other persistent strains can be isolated over the same periods [10–12]. Although several explanations have been proposed for the persistence of specific *L. monocytogenes* strains [13], the reasons are not fully understood. It has been postulated that *L. monocytogenes* strains persist due to opportunistic colonization of harborage sites in food processing facility premises and on equipment [13]. Alternatively, it has been suggested that the persistence of *L. monocytogenes* is linked to its ability to produce biofilms [14, 15]. Other studies using phenotypic methods have observed resistance to quaternary ammonium compounds (QAC) in persistent strains [16–19]. Recently, genetic factors associated with the QAC-resistance phenotype and resistance to benzalkonium chloride (BC), a QAC largely used in the food industry, were identified [17, 20–23]. These include the benzalkonium chloride resistance cassette *bcrABC* [21], in which *bcrAB* code for the small multidrug resistance protein family (SMR) transporters and *bcrC* codes for a transcriptional factor [23]. In addition, the *qacH* gene which codes for a SMR transporter associated with the export of benzalkonium chloride and acquired by the Tn*6188* transposon [19, 20, 24], and other QAC determinants originally observed in *Staphylococci* [25].

Although there is no consensus on the definition of a persistent strain, a persistent status should be considered when the same molecular type is isolated many times at different visits within the same processing plant for a defined period of time [13]. Therefore, identification of recurring highly genetically related isolates is the first step necessary in studying the association between resistance to BC and persistence. The persistence designation used in previous studies was based on pulsed-field gel electrophoresis (PFGE) typing [19, 24], an international standardized method for *L. monocytogenes* characterization. However, recent studies using whole-genome sequencing (WGS) enabling the core genome/whole genome MLST (cgMLST/wgMLST) analyses and single nucleotide variant (SNV) analyses have demonstrated that a better discriminatory power can be achieved [26, 27]. WGS-based typing methods may allow for better clustering of highly-related persistent isolates.

In this study, we set to characterize three groups of *L. monocytogenes* strains – two persistent and one sporadic – isolated from a pig slaughterhouse, with a particular emphasis on the BC resistance genotype/phenotype of these strains. We also performed complementary analyses to identify high quality single nucleotide variants (hqSNVs) and predict affected functions on the basis of these SNVs.

## Materials and methods

### Sampling, bacterial isolation, and pulsed field gel electrophoresis characterization

Sampling procedures, bacterial isolation and pulsed-field gel electrophoresis (PFGE) typing were performed according to Lariviere-Gauthier et al., [12]. Briefly, bacterial strains were isolated from repeated environmental sampling at a pork slaughterhouse and cutting facility. The samples used in this study were collected during three separate visits over a one-month period, with an interval of 2 weeks between visits 1 and 2 and 1 week between visits 2 and 3. Samples were collected using pre-moistened swabs, after cleaning and sanitation procedures, on environmental surfaces in contact or not with the products, a list of surface types is detailed in Table 1. The disinfectant concentration used in this slaughterhouse, and specifically in cutting facility, was from 150 to 200 ppm.

**Table 1:** Strain origin and pulsotype (as determined by CDC PulseNet PFGE protocol) of isolates obtained during monitoring sampling of an industrial slaughterhouse over a four weeks period

Isolates	ST ID (PFGE type^a^)	Isolate characteristics^b^	Isolation dates (visit)	Origin
p960			2011-02-26 (1)	Viscera tank
p962			2011-02-26 (1)	Chilling door
p958		Persistent Group A	2011-02-26 (1)	Saw
p975	ST9 (16)		2011-03-19 (2)	Saw
p985			2011-03-27 (3)	Saw
p984			2011-03-27 (3)	Bleeding
p963				
p974				
p964	ST5 (1)		2011-02-26 (1)	
p966				
p967				
p965				
p968				
p971				meat conveyor
p972	ST5 (1)	Persistent Group B	2011-03-19 (2)	
p969				
p970				
p973				
p980				
p977				
p978	ST5 (1)		2011-03-27 (3)	
p979				
p981				
p982				
lis8316	ST5 (1)		2014-01-12	
p959	ST5(10)		2011-02-26 (1)	Line
p961	ST9(18)		2011-02-26 (1)	Chilling entry
p957	ST1(8)	Sporadic Group C	2011-02-26 (1)	Saw
p976	ST5(9)		2011-03-19 (2)	Floor
p983	ST6(6)		2011-03-27 (3)	Door

^a^PFGE type corresponds to the Chaire de recherche en salubrité des viandes (CRSV) nomenclature [12]

^b^Persistent vs sporadic groups determined by PFGE

Another strain, Lis8316 belonging to the group B was isolated 3 years after in the same slaughterhouse.

Detection of *Listeria monocytogenes* was performed using Health Canada’s MFHPB-30 based-method as described previously [12], briefly, two enrichment broths were used. The first enrichment was conducted in University of Vermont medium 1 broth (UVM-1; Lab M, Heywood, United Kingdom) for 24 h at 30 C°, and the second in Fraser broth (Lab M, United Kingdom) for 48 h at 37 C°. Selective Listeria Ottavani and Agosti (ALOA; AES Chemunex, Bruz, France) agar was used to isolate *L. monocytogenes* strains from the two broths and the identification of isolates were based on the use of carbohydrates, Christie-Atkins-Munch-Petersen (CAMP test), hemolysis and motility.

The pulsed field gel electrophoresis characterization of isolates was based on the Center disease and control center (CDC) PulseNet protocol [28]. Pattern analysis was performed using Bionumerics software (version 6.5, Applied Maths, Kortrijk, Belgium) and strains were clustered with the unweighted pair group method with arithmetic means.

For whole genome sequencing, strains were selected according to their recurrence during the sampling period (transient vs persistent). PFGE type 1 and 16 belonging to the sequence type (ST) 5 and 9 respectively, were considered persistent since they were isolated at each visit and after cleaning and sanitation procedures (respectively named persistent B and A). PFGE types 6, 10, 9, 18, 8 respectively with a ST of 6, 5, 5, 9 and 1 were considered to be transient or sporadic strains since they were isolated only at one visit. The persistent group A and B and sporadic strains are phylogenetically unrelated, even if the sporadic strain p961 (pulsotype 18) showed a polymorphism of three different bands with the group A considering Apa1 and AscI restriction profiles. A PFGE profiles of a representative strains from the group A and B and p961 strain (pulsotype 18) were given in a Additional file 1: Figure S1. The numbers and characteristic selection of isolates are detailed in Table 1.

### DNA extraction and sequencing

One mL of Brain Heart Infusion (BHI) broth (Becton and Dickinson, Franklin Lakes, NJ, USA) was inoculated with a single colony of *L. monocytogenes* and incubated for 4 to 6 h at 37 °C. Bacterial cells were collected from 400 μL of culture by centrifuging at 15,000 x g for 2 min. Bacterial pellets were resuspended in 200 μL of lysis buffer containing 1% Triton X-100 (Sigma-Aldrich, ON, Canada) and 10 mg/mL lysozyme (Fisher scientific, ON, Canada) and incubated for 20 min at 37 °C. Genomic DNA (gDNA) was extracted using the Maxwell® 16 Cell LEV DNA Purification kit (Promega, Madison, WI) as recommended by the manufacturer. DNA was then quantified using the Quant-iT™ High-Sensitivity DNA Assay Kit (Life Technologies Inc., Burlington, ON). Sequencing libraries were constructed from 1 ng of gDNA using Nextera XT DNA Sample Preparation and Index Kits (Illumina, Inc., San Diego, CA) according to manufacturers’ instructions. Genomic sequencing was performed on the Illumina MiSeq Platform (Illumina, Inc.) using a 600-cycle MiSeq Reagent kit v3 (Illumina, Inc.).

### Genome and plasmid assembly and annotation

Reads were quality checked and de novo assembled using SPAdes 3.12.0 [29] with default settings. The resulting draft genomes were analyzed for assembly quality using Quast 4.1 [30]. For plasmid assembly, plasmidSPAdes [29] pipeline was used to assemble only the plasmid from the WGS data using the default settings. To identify the presence of large plasmid pLM80 harboring the bcrABC cassette, the resulting plasmid assemblies were blasted online using the basic local alignment search tool (Blastn) from the National Center for Biotechnology Information’s (NCBI) website (https://blast.ncbi.nlm.nih.gov/Blast.cgi) with default settings using the nucleotide collection (nr/nt) database.

### Core genome MLST (cgMLST) characterization

cgMLST was conducted using a genome based characterization method developed by Moura et al. [26]. The assembled 30 genomes were submitted to the Institut Pasteur’s website (http://bigsdb.pasteur.fr/listeria/listeria.html) for cgMLST typing using 1748 loci and a cgMLST type (CT) was defined as a group of cgMLST profiles that differ by up to seven allelic mismatches out of 1748 loci.

### High quality core genome single nucleotide variant (hqSNV) analysis

The paired end reads of closely related isolates were quality trimmed and analyzed for hqSNV detection by using the SNVPhyl core genome hqSNV pipeline (https://github.com/apetkau/core-phylogenomics) [31], available as a tool shed in Galaxy (https://toolshed.g2.bx.psu.edu/). Briefly, the reference genomes were analyzed with PHASTER [32, 33] for identifying phage regions. The paired end reads were mapped against the reference genomes using SMALT v0.7.5 (http://www.sanger.ac.uk/science/tools/smalt-0). The FreeBayes 0.9.20 was used to call the high-quality variants with a minimum depth coverage of 15 and a minimum mapping quality of 30. The minimum SNV abundance ratio was adjusted at 0.75. The uncalled positions were analyzed for sufficient coverage with the BCFtools that came in the SAMtools package. Reference genomes EGD-e (NCBI reference number NC003210.1) and 1/2b str 10–0810 (NCBI reference number NZ_CP007168.1) were used for groups A and B respectively, according to their phylogenetically relatedness throughout all analyses.

### SNV analysis

The resulting variants (hqSNVs) were annotated and filtered to the reference genome *Listeria monocytogenes* EGD-e (NC003210.1) with snpEff and snpSift program respectively [34] for the prediction of affected functions based on the identification of genes in which the mutations were found. In this study, only the high impact hqSNVs were taken into consideration (i.e. SNVs leading to a stop gained, start loss, or a frameshift).

### Screening of benzalkonium chloride resistance genes in *L. monocytogenes* strains

Identification of genetic determinants for resistance was conducted at the Institut Pasteur, according to their established protocol [26]. Briefly, a set of resistance genes was identified based on previous studies [35–37] and PATRIC, a public resistance gene database [38]. A BLASTn algorithm was used to identify the resistant genes with a minimum nucleotide identity of 70% and alignment length coverage of 70%. The results were represented in a presence-absence heatmap using the phandango visualization tool [39].

### Minimal inhibitory concentration of BC and PCR screening for *bcrABC*

To determine if the persistent groups were resistant to BC, Minimal inhibitory concentrations (MICs) were determined according to the clinical and laboratory standards institute (CLSI) guidelines with minor modifications. Briefly, tryptic soy broth with yeast extract (TSBYE) supplied with BC (Sigma-Aldrich, ON, Canada) at a final concentration covering a range from 0 to 200 ppm was inoculated with 10^− 5^ CFU from an overnight culture. The plates were incubated at 30 °C for 48 h. The MIC was defined as the lowest concentration at which a growth inhibition was observed. The assay was conducted in triplicate.

PCR was used to confirm the presence of *bcrABC* genes. DNA was extracted from sporadic and persistent strains using Chelex (Biorad, Mississauga, ON, CA) as follows: isolates were plated on blood sheep agar and colonies were picked and resuspended in 50 μL of Chelex at 0.6% (*w*/*v*). The suspensions were heated first at 55 °C for 30 min and then at 98 °C for 15 min and were vortexed between steps. The solutions were centrifuged at 12000 rpm and the upper phase containing DNA was transferred to a clean tube and stored at 4 °C for quick use. The PCR parameters consisted of an initial denaturation of 98 °C for 30 s followed by 30 cycles of denaturation at 98 °C for 30 s, annealing at 60 °C for 40 s, elongation at 72 °C for 1.30 min, and final elongation at 72 °C for 5 min. The primers used here were designed in a previous study [21, 40].

### Biofilm formation ability

Isolates from all groups were tested for biofilm formation ability using the microtiter plate assay in the presence and absence of BC (Sigma-Aldrich, ON, Canada), briefly, an overnight culture in tryptic soy broth supplemented with 0.6% (*w*/*v*) of yeast extract was inoculated to BHI (Becton and Dickinsen, Franklin Lakes, NJ, USA) supplemented or not with 3.125 and 0,78 ppm of BC (at final concentration) at 1/1000. 100 μL of the inoculated BHI was distributed into each well of 96 well microplates (Costar® 3370; Corning, NY, USA) and incubated at 30 °C for 48 h. The supernatant was discarded from the mature biofilm and this latter washed to eliminate planktonic cells before stained with Cristal Violet (CV) 0.1% (w/v) for 20 min and washed two times. Ethanol 95% was used to solubilize CV within biofilm and Optical density (Od) was taken at 595 nm. Experiment was repeated in triplicate at different days.

### Statistical analysis

To determine if there were differences in biofilm formation capacity between strains from the three groups in the presence and absence of BC, linear mixed model was performed on the data of groups (A, B, C) and treatments (control, 3.125 and 0.78 ppm) as fixed factors and isolate as random factor. A series of a priori contrasts were conducted to compare the mean in the control groups with the mean in the other two treatments in each group and to compare the mean in each group for each treatment. Alpha level was adjusted with Benjamini-Hochberg procedure. Results were significantly different if *p* < 0.05.

## Results

A total of 30 *L. monocytogenes* strains was isolated: 25 were persistent, present during all visits, and 5 were sporadic, present during a single visit. The 25 persistent isolates were sub-divided into 2 Groups, A, 6 isolates, and B, 19 isolates, based on their pulsotypes. The 5 sporadic isolates were assigned to Group C (Table 1).

**Fig. 1:**
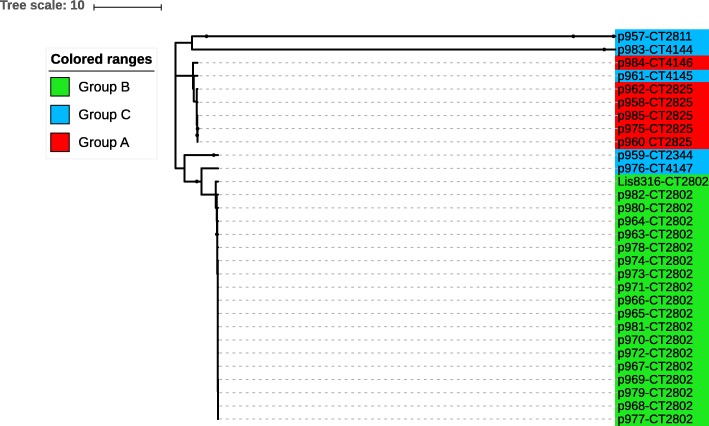
Phylogeny reconstruction of the persistent groups A, B and the sporadic strains group C isolated from an industrial slaughterhouse over a 4 weeks period; the tree was based on the cgMLST allelic profile distances. The interactive tree of life (ITOL) was used to visualize the tree [57]

The cgMLST analysis showed eight cgMLST types (CT) corresponding to the six Pulsotypes. It is noteworthy that all of the CTs observed in our study were new in the BIGSdb database of the Institut Pasteur (at time of analysis). All of the strains from the group B (pulsotype 1) belonged to the new CT2802 whereas the strains from group A (pulsotype 16) showed two different CTs, with one strain belonging to CT4145 and the rest of the group to CT2825 (Fig. 1). Interestingly, the sporadic strain p961 (pulsotype 18) from sporadic strains group was closely related to persistent group A, and sporadic strains p959 and p976 were closely related to group B (Fig. 1).

**Fig. 2:**
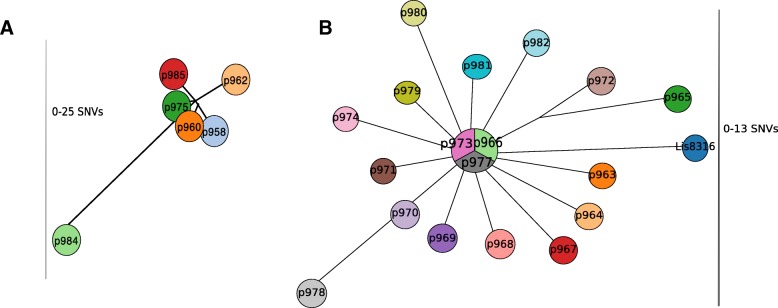
Minimum spanning tree based on high quality core genome SNV positions identified amongst 6 genomes over 80% of the reference genome for the group A (**a**), and amongst 19 genomes over 80% of the reference genome for the group B (**b**). The visualization of the MST was done using GrapeTree [58]

The SNVphyl analysis showed some differences in hqSNV’s numbers within the persistent strains: with 0 to 25 hqSNVs in group A and 0 to 13 hqSNVs in group B. Thus, in group A, only the p984 strain exhibited a high number of hqSNVSs (25) and was more distant from the remaining strains (Fig. 2a). Almost all the strains from group B harbored 4 hqSNV differences or fewer, except P978, p965 and Lis8316 strains which had more hqSNV differences compared to the rest of group B. This was reflected by the close positions in the phylogenetic tree (Fig. 2b).

### SNVs confined mostly to prophage regions

**Fig. 3:**
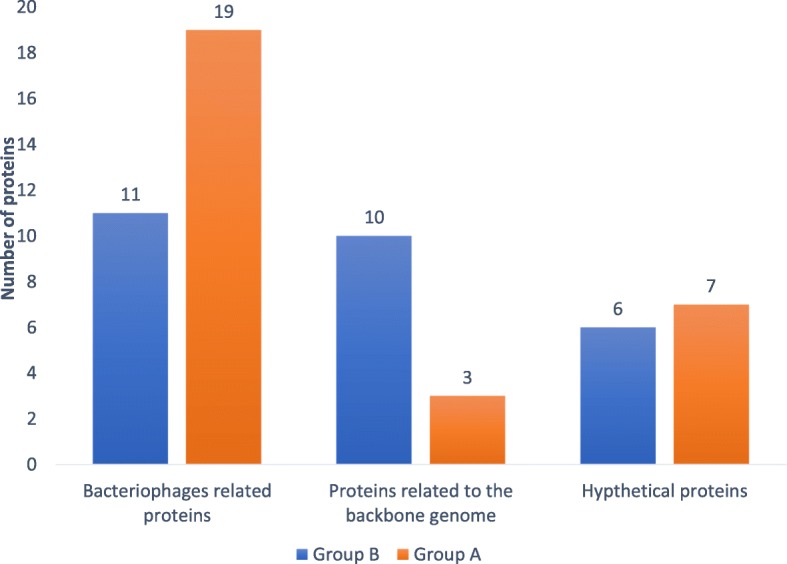
Number of SNVs in *Listeria monocytogenes* strains isolated from an industrial slaughterhouse over fourweeks according to their annotations and function predictions using the snpEff in the persistent groups A and B. All the inferred functions to the known bacteriophage proteins were grouped in “Bacteriophage proteins”, all hypothetical proteins were grouped in one cluster, and the other affected functions related to the backbone genome of *L. monocytogenes* were grouped

To study different functions affected by mutations, hqSNVs were analyzed with the snpEff program. The inferred function of impacted proteins by the hqSNVs were mostly related to bacteriophage proteins, particularly in group A (Fig. 3). Most of the hqSNVs were found within phage A118 (Table 2A). Furthermore, the position of these phages within the genome was the same between the unrelated persistent groups A and B (Table 2A and B). There were 8 hqSNVs that were detected in genes coding for known functions of the *L. monocytogenes* core genome in group B, but five out of these eight were present in only one to four strains (Table 2B). Otherwise, in group B, a SNV in the *comk* gene was identified at a position that resulted in a disruption of this gene due to loss of a stop codon (Table 2B). A stop codon in the *inlA* gene was detected as well as the SNVs, and this was confirmed by Sanger sequencing of the *internalin A* gene (*inlA)* in group B strains in a previous study [12]. These SNVs lead to a premature stop codon (PMSC). Interestingly, another SNV was detected in an *internalin B* gene (*inlB*) confirmed by BLASTn. The rest of the SNVs were detected in genes coding for hypothetical proteins. Among them, many shared similarities with the A118 phage proteins (Additional file 2: Table S1).

**Table 2:** Composition of the annotated high impact SNVs in the persistent groups A (Table A) and B (Table B) in *Listeria monocytogenes* strains isolated from an industrial slaughterhouse over four weeks with their position and the altered nucleotide according to the references of the respective persistent groups

SNVs	REF-ALT^a^	Position	Protein inference
A)			
H28	A-T	2,365,617	holin
H2	C-A	2,366,926	protein gp20
H6	A-C	2,370,180	Protein gp17
H11	G-T	2,371,838	putative tape-measure
H12	G-T	2,376,360	Protein gp15
H13	A-T	2,378,030	Portein gp11
H7	G-C	2,383,240	putative portal protein
H8	G-C	2,387,299	Protein gp66
H23	T-C	2,396,331	Protein gp43
H14	C-A	1,134,999	Cadmium resistance protein
H20	T-A	2,268,215	putative peptidoglycan bound protein (LPXTG motif)
H3	G-A	2,397,250	Hypothetical protein
H21	G-A	2,398,304	Hypothetical protein
H4	C-A	2,399,019	Hypothetical protein
H22	T-A	2,435,024	Hypothetical protein
H24	T-C	2,725,852	Hypothetical protein
H29	C-T	2,806,268	Hypothetical protein
H30	C-T	2,862,889	Hypothetical protein
H25	A-G	2,928,670	Hypothetical protein
H9	G-C	2,388,916	Hypothetical protein
H5	T-C	2,389,387	Hypothetical protein
H19	G-A	1,062,031	Hypothetical protein
H26	G-T	1,106,182	Hypothetical protein
H27	G-T	1,683,477	Hypothetical protein
H15	A-T	2,363,489	Hypothetical protein
B)			
H1	T-C	13,003,993	DUF3310 domain-containingprotein
H2	G-T	1,307,219	Phage tail measure protein
H6	T-A	1,314,894	Phage tail tape measure protein
H23	T-C	1,300,393	VRR-NUC domain-containing protein
H11	A-T	2,467,036	Phage antirepressor Ant
H12	C-G	2,473,719	recombinase family protein
H15	G-A	1,323,444	DUF3800 domain-containing protein
H16	T-A	447,408	Polysaccharide deacetylase
H17	T-A	463,360	Internalin A
H18	T-A	465,225	Internalin
H19	C-A	2,932,396	6-phospho-beta-glucosidase
H21	T-A	1,146,752	dTDP-4-dehydrorhamnose reductase
H22	T-A	2,347,583	Oligoendopeptidase F
H26	G-T	431,637	alpha-mannosidase
H27	G-T	972,616	GntR family transcriptional regulator
H8	T-G	2,432,776	Competence protein Comk
H13	G-T	1,307,219	Peptidase
H5	G-A	2,465,545	Hypothetical protein
H9	G-T	2,445,638	Hypothetical protein
H20	G-A	2,940,879	Hypothetical protein
H24	A-C	2,458,383	Hypothetical protein
H25	A-G	79,636	Hypothetical protein
H28	A-T	1,430,257	Hypothetical protein
H29	A-T	444,637	Hypothetical protein
H10	G-T	2,461,077	hypothetical protein
H14	G-T	2,445,638	Hypothetical protein

^a^*REF-ALT* Reference-Alteration: Definition of the nucleotides present in the reference genome and their substitution after the mutation in the genomes of this study

### Presence of *bcrABC* in a persistent strains group B

**Fig. 4:**
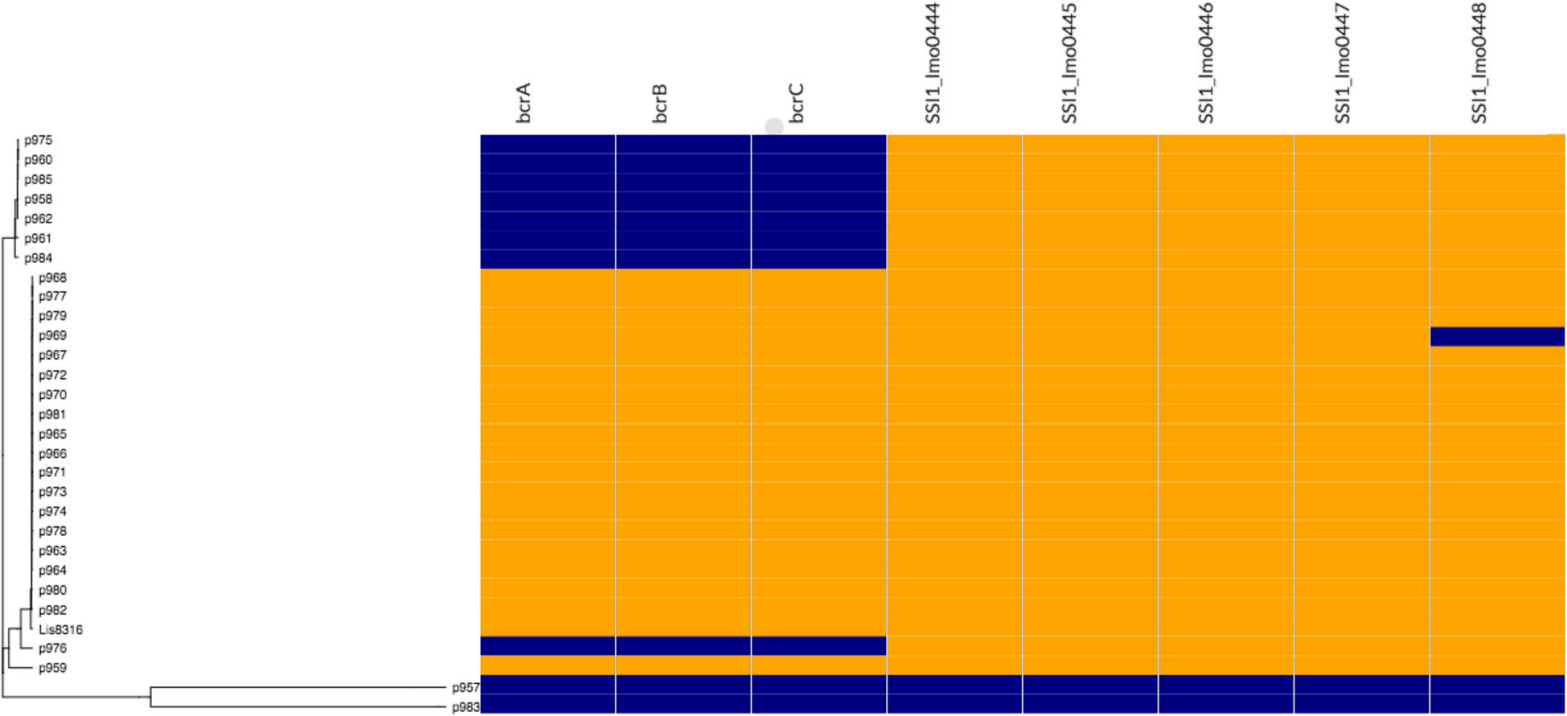
Phylogenetic distribution of the resistance genes *bcrABC* and stress response genes (ssi-1) in *Listeria monocytogenes* strains isolated from an industrial slaughterhouse over four weeks; the presence-absence heatmap of the *bcrABC* and *ssi-1* in the persistent and sporadic groups are shown in orange when present and blue when absent

**Fig. 5:**
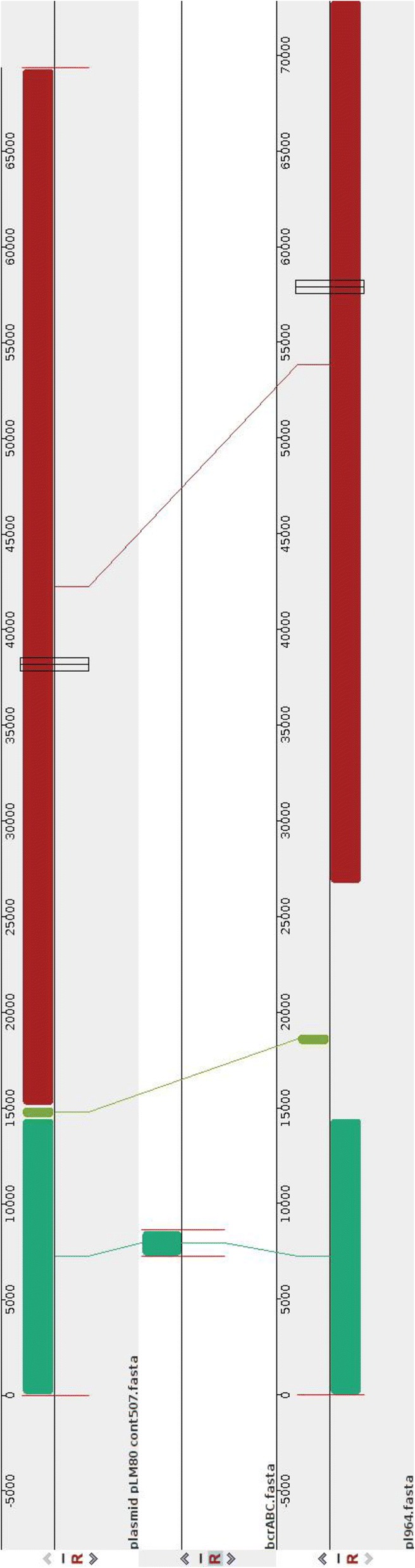
Multiple sequence alignment of the *bcrABC* genes (accession number JX023284.1) and pLM80 cont 507 (accession number: AADR01000010) with a large plasmid identified in in persistent group B as shown in selected strain p964 (p964) and in a closely related strain Lis8316, isolated three years after the isolation of the rest group B strains

In order to study the presence or absence of genes associated with the persistence phenotype, identification of accessory genes was conducted on the persistent groups as well as on the five different sporadic strains isolated from the same slaughterhouse. Our results showed the presence of *bcrABC*, a resistance gene cassette to BC, a commonly used agri-food sanitizers [17, 20–23]. More importantly, this genetic determinant was present in only persistent group B and in its closely related sporadic strain p959, while the stress response island genes (*ssi1*) were present in all strains except p957 and p983 (Fig. 4). The *bcrABC* cassette was identified in a plasmid with 80 kbp in all the persistent strain group B with 100% similarity with plasmid pLM80-cont 507, previously described [41] with accession number of AADR01000010 (Fig. 5). In addition to *bcrABC*, this plasmid was identified as a vector for cadmium and arsenic resistance as well [41]. To confirm the presence of the *bcrABC* genes in only the group B, a PCR was conducted in all studied isolates. The PCR confirmed the presence of the *bcrABC* cassette in the group B and its absence in the group A and in sporadic strains (Table 3). Furthermore, using the CMI test, we observed growth in all isolates from the group B at 6.25 ppm of BC, indicating decrease of sensitivity to this compound, while the rest of isolates from the group A and C did not grow at this concentration (Table 3).

**Table 3:** In silico and phenotypic characteristics of *L. monocytogenes* strains from group A, B, and C isolated from an industrial slaughterhouse over a four weeks or three years (for Lis8216) period; In silico identification of determinants for persistence. Phenotypic determination of resistance to BC in *L. monocytogenes* strains isolated: Minimal inhibitory concentrations of all isolates. PCR screening test to identify the *bcrABC* cassette in the studied strains

Genetic determinants	Strains		
	Group A	Group B	Group C
In silico identification			
bcrABC resistance genes	–	+	–
*Stress survival islet SSI-1*	+	+	+
*comk prophages*	–	+	–
*Presence of pLM80 plasmid*	–	+	–
Phenotypic confirmation			
*bcrABC* detection by PCR	–	+	–
Minimal inhibitory concentration	–	+	–

### BC effect on biofilm ability is strain and concentration dependent

**Fig. 6:**
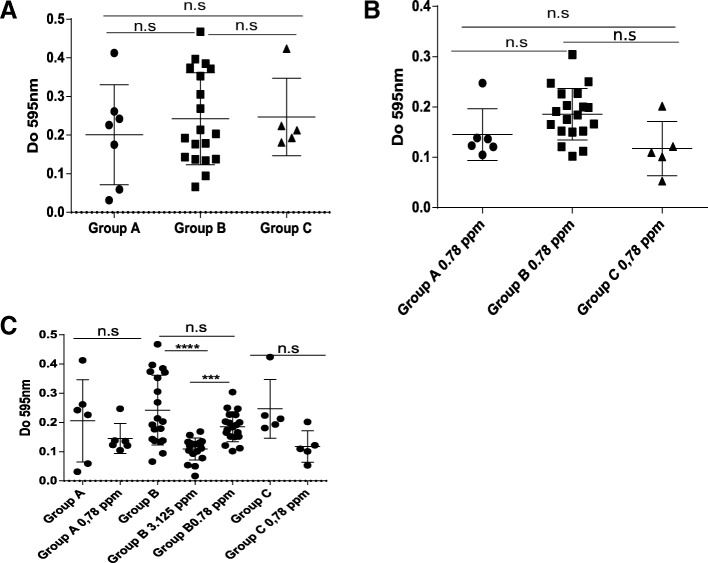
Biofilm formation ability of *L. monocytogenes* strains from persistent groups A, B and sporadic group C isolated from a pig slaughterhouse at three or four visits (for group B). **a**) Comparison of biofilm formation ability between the group A, B and C in the absence of BC. **b**) Comparison of biofilm formation ability between the group A, B and C in the presence of BC. **c**) Effect of BC at 3.125 and 0.78 ppm on biofilm formation capacity of persistent strains from the group A and B and the sporadic strains C. Significance, n.s: non-significant, ****: *p* < 0.0001, ***: *p* = 0.0014.

In order to investigate the ability of biofilm formation by strains from the persistent group A, B and the sporadic strains in the presence and absence of BC, microtiter plate assay for biofilm growth was performed on all isolates. Since only strains from group B grew at 3.125 ppm and strains from groups A and C grew in the presence of 0.78 ppm of BC, comparisons were done at the latter concentration. Results showed no significant difference between isolates from the three groups in absence or presence of BC (Fig. 6a and b, respectively), in fact all the strains were able to form biofilm and seem to have an equal ability for biofilm formation. To study if BC affected the biofilm formation of strains in each group, biofilms formed in the presence of BC at 3.125 ppm (for group B strains) and 0,78 ppm were compared to the biofilms formed in the absence of BC. Results showed that biofilms formed in presence of BC decreased for all groups at all concentrations of BC, but the differences were not significant for group A and C strains grown in the presence of a sublethal concentration of 0.78 ppm compared to the biofilms grown in the absence of BC (Fig. 6c). In contrast, for group B strains, biofilm formation in the presence of BC at a sublethal concentration of 3.125 ppm was significantly lower than growth in the absence of BC (Fig. 6c).

## Discussion

The aim of this work was to identify genetic determinants that could be associated with persistence of *L. monocytogenes* strains within the slaughterhouse environment. We were especially interested in the potential role of genetic determinants related to resistance to quaternary ammonium, an antimicrobial compound commonly used as a disinfectant in slaughterhouses.

The persistence phenotype of some *L. monocytogenes* strains has been previously reported [3, 9, 13], however, the reason why these strains can persist in the food processing environment is yet to be fully understood [7]. Here, we describe genomic characteristics of two unrelated persistent groups of strains isolated from slaughterhouse environments at three different times. Although our sampling interval was relatively short, we believe that the isolation of the same CTs at the same location after daily cleaning and sanitation procedures, is a sufficient parameter to attribute the persistence character to our isolates. Furthermore, a sampling completed 3 years after our sampling for this study at the same location allowed us to isolate the same CT from the group B (Lis8316).

The cgMLST and the SNVphyl analysis confirmed the close genetic relatedness between isolates of pulsotype 1 from group B belonging to ST5 and clonal complex (CC) CC5, a clonal group involved in a variety of food products and processing environments contamination as well as in at least two outbreak listeriosis in the United states [42]. Two different CTs, CT2825 and CT4146 were distinguished in group A strains (pulsotype 16) leading to improved discriminatory power, as reported in previous studies [26, 43]. These CTs belonged to ST9 which was described previously as highly prevalent in the food associated *L. monocytogenes* strains [44–46]. The SNVphyl analysis showed the presence of 0 to 13 hqSNV differences in the persistent groups, except for strain p984, in group A, where the number of different hqSNVs reached 25 relatives to the rest of the strains. This result was in accordance with cgMLST typing as this strain had a different CT from the rest of group A. The number of hqSNVs did not vary significantly between each period and between strains within the two persistent groups, indicating that the strains were closely related. Indeed, there were no differences between them based on the number of SNVs. Such results are in accordance with other studies that observed limited accumulation of SNVs between two closely related strains isolated from a food processing plant at a 12 year interval [3]. In the current study, a great proportion of SNVs were confined to prophage regions in the genomes and there were very few biologically significant mutations affecting the core genome. Thus, while previous studies showed that prophage insertion into the *L. monocytogenes* genome was the most important element contributing to the diversification of the strains [47], here we demonstrated in silico that the genes most affected by non-synonymous SNVs were also related to the prophage genes. These findings support the largely recognized view of the high stability of the *L. monocytogenes* genome [26, 37, 47, 48]. The presence of the *comk* prophage in the group B strains is in accordance with the persistence character of these strains and the association of the ST5 strains to food processing environment as described in previous study [49]. This study allowed the identification of *bcrABC* in persistent strains group B. These results complement the Ortiz et al. study [19] where they found the *tn6188* transposon, which is known to serve as a vector for the *qacC* resistance gene transfer in persistent strains. Efflux pump *bcrABC* was described in *L. monocytogenes* for the first time by Elhanafi et al. in a large *Listeria monocytogenes* plasmid, pLM80 [21]. This plasmid was also previously identified as the vector for cadmium and arsenic resistance determinants in *L. monocytogenes* [41].

Since then, many studies found the *bcrABC* in strains isolated from retail foods [23, 50, 51]. The presence of *bcrABC* in strain Lis8216, isolated within a 3-years interval, may indicate the stability of the plasmid harboring this gene in environments where BC is commonly used. Moreover, persistent group B was isolated from conveyor surfaces, which have the greatest exposure to BC. However, in our analysis, if these strains were confirmed as less sensitive to BC treatments, they should still be considered as sensitive to BC concentrations used in the slaughterhouses where the sampling was done. This apparent paradox was previously reported [17, 25, 52] and was attributed to a dilution effect during sanitation procedures on wet surfaces, leading to sub-inhibitory concentrations of the disinfectants as reported previously [53]. Persistent group A did not harbor the *bcrABC* cassette, and strains from this group were more sensitive to BC as compared to the ones in group B. Therefore, group A isolates were susceptible to BC. It is noteworthy that these strains were collected from equipment (e.g., saws), places known to be hard to clean, one may suppose that strains residing in such areas could not be exposed frequently to disinfectant. It is therefore easy to hypothesize that strains from group A did not submit pressure selection for *bcrABC* acquisition. Results for biofilm formation ability showed no significant differences between strains from persistent and non-persistent groups and all isolates were able to form a 48 h mature biofilm. Similar results were shown in previous studies where persistent and non-persistent *L. monocytogenes* strains isolated from different sources showed an equal biofilm formation capacity [54]. Same results were obtained from environmental and food product strains in other recent studies where authors suggest that biofilm formation could not be a determinant for persistence [55] or the persistence of studied *L. monocytogenes* strains was not linked to biofilm formation ability and other determinants should be investigated [56]. The presence of BC at 0.78 ppm during biofilm formation seems not to affect biofilm amount between the three groups, and when strains were compared for their ability to form biofilm in the presence and absence of BC, only isolates from group B seemed to be affected by the sublethal concentration of BC at 3.125 ppm, which may indicate the strain-specific variable responsiveness to the effect of BC on biofilm formation, and it appears that BC-resistant strains produced significantly less biofilm than susceptible strains as reported previously [24]. It is not understood why BC-resistant *L. monocytogenes* strains had less ability to form biofilm in the presence of BC, though previous investigators suggested that modification of cellular morphology, phospholipids and fatty acids could be involved in biofilm formation [24].

Further studies using a greater number of unrelated persistent and non persistent strains should be conducted in different conditions, particularly those which mimic the slaughterhouse environment.

Our findings support the hypothesis that persistence, in the case of the strains from the group A, was related to biofilm formation and/or ecological niches formation in hard-to-clean places. Indeed, some authors suggested that persistence could be related to colonization of ecological niches less accessible during sanitation procedures as reported in previous reviews by Carpentier and Cerf (2011) and Ferreira et al. (2014) where authors concluded that there was no association between persistence and any phenotypic characteristics like biofilm formation and resistance to QAC [7, 13]. While we cannot exclude that some environmental sites were less efficiently cleaned, our findings support that there is not always only one determinant explaining the persistence of *L. monocytogenes* strains and that this phenomenon could be due to multiple factors facilitating the survival of this bacterium. This could explain why some studies focusing on only one determinant, like resistance to QAC, could not find a causal effect with persistence [9, 19]. We propose that the localized environmental conditions in which strains were isolated in the food processing plant could give clues to identify which determinant(s) enhances the strain’s survival. Thus, isolates collected from a conveyor known to be the most exposed surfaces to QAC disinfectants may benefit from QAC resistance while isolates recovered from difficult to clean surfaces may benefit from better biofilm formation phenotypes. We believe that persistent strains possess at least one, but most likely many persistence-associated determinants leading to survival in their specific environment conditions.

## Conclusion

In this study, we described the distribution of non-synonymous SNVs in persistent *L. monocytogenes* genomes from a food processing environment and found evidence that the presence of genetic determinants associated with resistance to BC could be linked to the persistence of *L. monocytogenes* strains. The ability to differentiate between sporadic and persistent environmental contamination events using the methods described will be a key element in guiding risk management actions, such as determining the type of sanitary interventions (e.g., aggressive versus standard) required to mitigate identified hazards and adapt cleaning and sanitation procedures to avoid recurrent food contamination.

## Additional files

Additional file 1: **Figure S1.** Pulsed field gel electrophoresis profile of AscI and Apa1 restriction enzymes in *L.* 20 *monocytogenes* strains isolated from a slaughterhouse over a four weeks period. (PDF 144 kb)

Additional file 2: **Table S1.** complete annotation of the high impact SNV and function prediction for the group A (spreedsheet group A) and group B (spreed sheet group B). (XLSX 14 kb)

Additional file 3: **Table S2.** PRJNA433177 Assembly Details and Institut Pasteur IDof the 29 *Listeria monocytogenes* strains isolated from an industrial slaughterhouse over a 4 weeks period. (XLSX 11 kb)

## Abbreviations

BC: Benzalkonium chloride
BHI: Brain heart infusion
CAMP: Christie-Atkins-Munch-Petersen
CDC: Center disease and control center
CLSI: Clinical and laboratory standards institute
CT: cgMLST Type
hqSNV: High quality single nucleotide variant
MICs: Minimal inhibitory concentrations
NCBI: National Center for Biotechnology Information
PFGE: Pulsed field gel electrophoresis
PMSC: Premature stop codon
QAC: Quaternary ammonium compound
SMR: Small multidrug resistance
SNV: Single nucleotide variant
ST: Sequence type
TSBYE: Triptic soy broth yeast extract
UVM: University of Vermont Medium
WGS: Whole genome sequencing
