# Supplementary material for: Genomic characterization of Listeria monocytogenes isolates reveals that their persistence in a pig slaughterhouse is linked to the presence of benzalkonium chloride resistance genes
Source: BMC Microbiol. 2018 Dec 20;18:220. doi: 10.1186/s12866-018-1363-9 (PMC6302515; doi:10.1186/s12866-018-1363-9)
Supplement: Supplementary file 1 — Figure S1. Pulsed field gel electrophoresis profile of AscI and Apa1 restriction enzymes in L. 20 monocytogenes strains isolated from a slaughterhouse over a four weeks period. (PDF 144 kb) [file 12866_2018_1363_MOESM1_ESM.pdf]

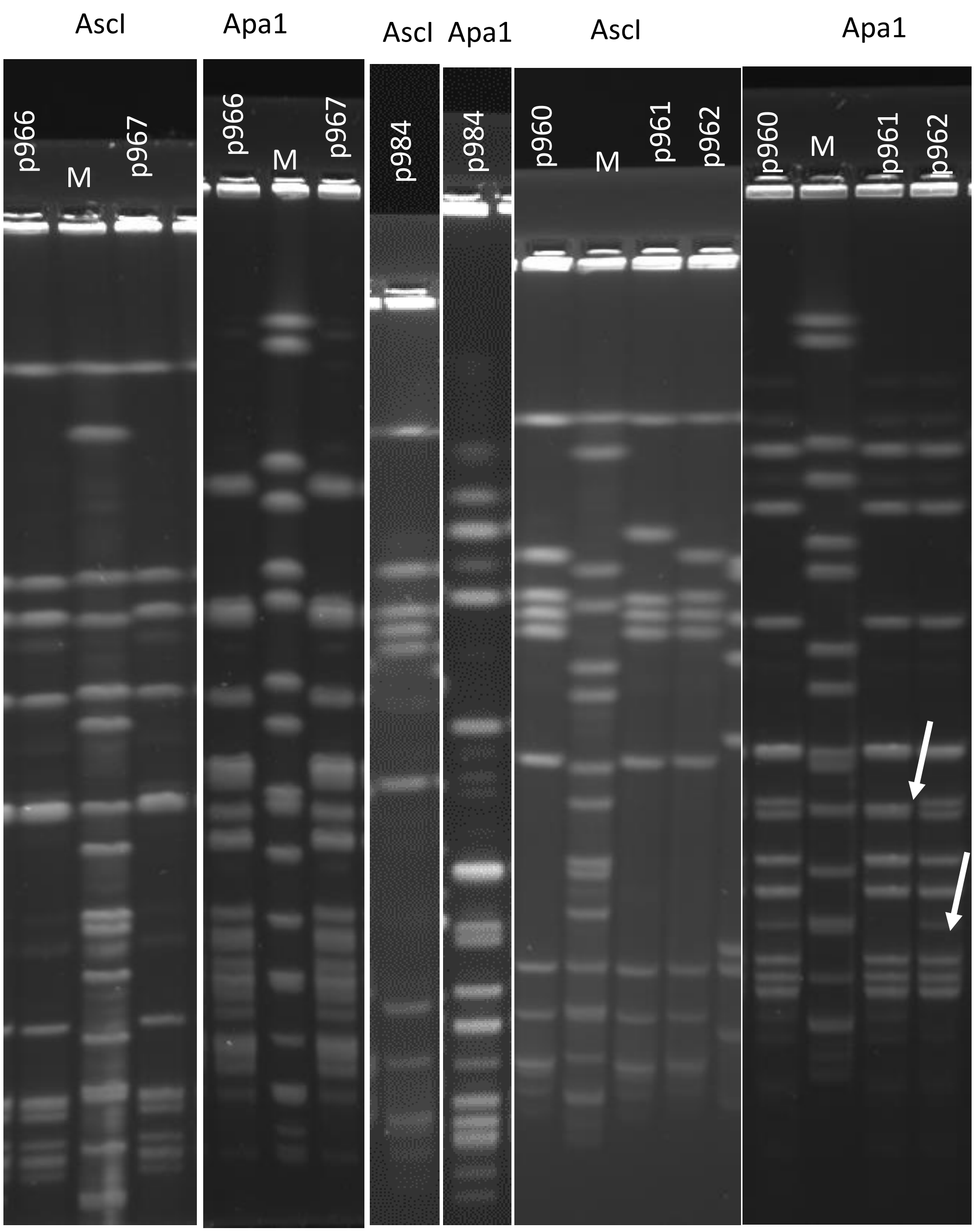

-Supplementary Figure S1

Pulsed field gel electrophoresis profile of AscI and Apa1 restriction enzymes in *L. monocytogenes* strains isolated from a slaughterhouse over a four weeks period.
